# Faith and Practice: How Religious Beliefs Affect a Music Therapist's Clinical Identity and Practice

**DOI:** 10.1177/15423050241291562

**Published:** 2024-11-21

**Authors:** Victoria Di Giovanni, Heidi Ahonen

**Affiliations:** 8431 Wilfrid Laurier University, Waterloo, Canada

**Keywords:** Music therapy, religion, spirituality, discrimination

## Abstract

Although there has been considerable literature surrounding spirituality and its role in music therapy, there stands to be a gap surrounding religious faith and its effect on clinical practice by practitioners. This phenomenological study, conducted among accredited music therapists in Canada, examined the impact religious faith has on their clinical practice. The study found that religious devotion had more positive benefits than negative repercussions in the workplace among these professional practitioners.

## Introduction

The current music therapy literature has explored the connection between spirituality and music therapy clients (Notarangelo, 2019; Tsiris, 2017; Wlodarczyk, 2007). However, the exploration of religiously devout music therapists and how their religious beliefs affect their clinical practice is under-represented (Elwafi, 2011). The terms “spirituality” and “religion” are often used interchangeably, and although both are relevant, they seem to carry different connotations within the music therapy community and abroad (Arrey et al., 2016). Religion is a system of defined beliefs and practices related to sacredness and morality defined by a unified body, whereas spirituality pertains to personal development and connectedness to our surroundings and beyond (Durkheim, 1915; Tacey, 2004). In recent decades, affiliation with organized religion has declined. Instead, there has been a growing shift toward spirituality, which is often seen as more socially desirable (Jones & Elliott, 2016). Spirituality has emerged as a popular alternative to religion, especially among younger generations, with many identifying as spiritual but not religious (Pew Research Center, 2015; Zinnbauer et al., 1999). However, Van Niekerk (2018) explains, “Despite the criticism of religion, it appears to be tenacious, that it is here to stay. And despite the idea that the secularization thesis has been so prevalent, there is no fear that religion will collapse” (p. 7). With the established presence of organized religion and the growing social acceptability of spirituality, those who practice organized religions may be subject to unique challenges due to their affiliation and the ways in which their religious identity is incorporated in clinical practice. It is crucial for a harmonious workplace that we, as a community, strive to understand and empathize with these challenges, fostering a culture of compassion and consideration.

This study analyzed how religious beliefs affect a music therapist's clinical practice and relationships in the workplace. It also explored how forthcoming music therapists are about their religious identities and their perceived experiences of discrimination for doing so. The findings of this study underscore the importance of open dialogue and engagement in understanding and addressing these issues.

## Literature Review

Most research regarding the dynamics between music therapy and religion is quite limited, with the current literature focusing on the spiritual component of the Bonny Method of Guided Imagery and music therapy within palliative care (Elwafi, 2011). Religion in music therapy literature is commonly paired alongside spirituality or used interchangeably due to music's transcendent abilities. Elwafi (2011) found that when this occurred, the terms spirituality and religion were often used inconsistently. Spirituality pertains to personal development, and although there is a spiritual element in religion, religion follows a unified system of beliefs and practices under a governing body (Durkheim, 1915; Tacey, 2004). In this paper, the authors interchangeably use the terms spirituality and religion, though the emphasis is on the spiritual component of religious devotion.

According to Aigen (2008), spirituality has gained widespread acceptance among the music therapy community. He suggests the term spirituality is “agreeable” with the secular ethos of music therapy due to its progressive belief system. Others have made clear the spiritual factors that present themselves through music-making. For example, dos Santos and Brown (2021) argue that spirituality or religion can play significant roles in music therapy, and Notarangelo (2019) argues that music therapy is inherently spiritual. Further, elements of music therapy are non-tangible (i.e., receptive), similar to religious practice. Godwin (2023) describes music therapy as metaphysical and its effectiveness as reliant on the belief systems of the practitioners and service users. However, with these notions, there seems to be an apprehension towards the specific role of religious affiliation in a music therapist's practice independent from spirituality. Pek and Grocke (2016) state, “Despite an increasing awareness of spirituality in healthcare, there is little known in regards to the spiritual or religious beliefs of music therapists and how these beliefs may impact clinical practice” (p.1). Music can access, in a unique way, the transcendent elements present in both spirituality and religion. It is important to explore the contributions religious affiliation may bring to music therapy.

Elwafi (2011) conducted a study investigating the impact religious beliefs have on a music therapist's clinical identity and practice in the United States. This phenomenological study used heuristic and narrative inquiry as its methodological approaches. The purpose of the study was to examine the role religious faith has on music therapists’ clinical practice by investigating the experiences of four music therapists: two who practiced Islam, one Christianity, and one Judaism. Findings in the study concluded that the music therapists’ religious beliefs informed their clinical practice (i.e., their clinical practice became an extension of their religious practice through their conception of helping) and that all participants expressed some level of conflict between their religious affiliation and their clinical practice (i.e., the Jewish participant feeling temporary discomfort when clients mentioned Jesus in sessions). Further, the study revealed experiences of perceived discrimination by participants (i.e., a Muslim participant finding it more challenging to obtain a job while wearing her hijab during job interviews compared to not).

In Australia, a study was conducted by Pek and Grocke (2016) to investigate the relationship between music therapists and their spiritual beliefs among Australian music therapists. The study used qualitative and quantitative methods through a survey sent by the Australian Music Therapy Association to its members. With a response rate of 18.7%, the study found that most music therapists were affiliated with an organized religion or considered themselves spiritual. The majority of participants felt their faith greatly assisted them in their reflective practices as clinicians. However, many also expressed the belief that their religious views should be kept private from their clients.

Further, an international study by Tsiris (2017) investigated a systematic overview of music therapists’ perceptions of spirituality and its relevance to their clinical practice. The study used a mixed methods approach to collect data through a survey with 36 closed and open questions and included religious practice under the umbrella of spirituality. A total of 358 music therapists from 29 countries took part in the study and found 76% believed spirituality to be connected to their identity and work, 64% felt spirituality informs their practice, 62% found their practice informs their spirituality, and 91% felt that music therapy contributes to a client's spiritual well being (among other statistics).

End-of-life care seems to be a setting where spiritual beliefs are more explicit with clients in music therapy sessions (Potvin et al., 2020). Notarangelo (2019) lists spiritual care as a strength of music therapy as it aids in “acceptance of the moment, working with imperfection, transcending barriers, transforming perceived failures and connecting deeply” (Notarangelo, 2019, p. 101). Further, a study by Wlodarczyk (2007) examined the effect music therapy had on the spiritual wellness of patients in hospice care. The study revealed that music therapy sessions stimulated more discussion of spiritual material than strictly verbal sessions. The results indicated that spirituality serves as an essential topic with terminally ill patients and that music therapy can be used as an effective tool in addressing the complexities experienced at the end of life.

With spirituality being a potential component in music therapy sessions, it is a controversial topic whether a clinician should disclose their religious affiliation with clients or wear any religious attire during the sessions. Zeiger and Lewis (1998) suggest this can create barriers to the therapeutic process. If a therapist does choose to wear visible religious symbols, they should expect to be stereotyped and must accept the consequences that follow. Zeiger and Lewis state:

The religious therapist must be aware of transference, countertransference, resistance, and proper management when considering the consequences of wearing religious attire, much the same way he or she would need to be aware of these issues when considering actively incorporating religious assessment into treatment. (p. 423)

Zeiger and Lewis take the traditional stance that religion and the therapeutic relationship should remain separate. The authors explain that the therapeutic process should be secular, and although religious content can be explored in sessions, the stance of the therapist should remain subjective and detached. A similar view is expressed by Fayek (2004), who argues that his ability to separate religious identity from clinical practice enhances his effectiveness as a psychoanalyst. However, a study by Captari et al. (2018) found that integrating patients’ religious and spiritual beliefs in clinical work improved overall functioning compared to the groups that did not have spiritual integration. Patients in the integrated group experienced a reduction in psychological distress and enhanced spiritual well-being. Furthermore, Potvin et al. (2020) suggests that when therapists disclose their spiritual beliefs, it can benefit clients by validating their experiences and be a point of connection. When used with the client's benefit in mind, it seems integrating spiritual beliefs in clinical work could be a tool to strengthen the therapeutic relationship.

## Methodology

The following pivotal questions were investigated: (1.) How do a music therapist's religious beliefs affect their clinical identity and practice? (2.) How comfortable do music therapists feel sharing their religious identities with their colleagues, peers and clients? (3.) Have music therapists experienced discrimination in the workplace for having their religious identities known?

This qualitative study, conducted in collaboration with music therapists who consider themselves religiously devout, utilized a heuristic and narrative inquiry within a phenomenological framework (Clandinin & Connelly, 2000; Moustakas, 1990). Their experiences and anecdotes offered invaluable insight into their clinical lives. Utilizing the framework established by Van Manen (1990), this phenomenological approach included descriptive and interpretive characteristics. The aim of using this approach was to gain an understanding of the lived experiences of the music therapists and their relationship with their religious affiliation as a clinician. A mix of quantitative and qualitative data was collected through a survey sent by the Canadian Association of Music Therapists (CAMT) inquiring about members’ religious practices. Based on the survey responses, select music therapists were contacted to participate in in-depth, semi-structured interviews to explore their survey answers further. Interviews were conducted over video conference and recorded to later be transcribed. The thematic aspects uncovered from the interviews were analyzed through the interpretive hermeneutic approach, aiming to understand the data as a whole and capture individual nodes of meaning (Van Manen, 1990). The identified themes became the source of reflection to interpret and understand its wholeness (Van Manen, 1990). Lastly, the research paradigm for this study was a constructivist-interpretive design using relativist ontology (Schwandt, 1994). This study aimed to understand the lived experiences of religiously devout music therapists, and in doing so, multiple realities were analyzed. By reconstructing the lived experiences of the interviewed music therapists, the hope was that multiple realities from diverse backgrounds could paint a fuller picture of religion's role in clinical practice and its relationship with colleagues, peers and clients. Such realities were constructed based on their lived experiences and used to further the understanding of religious faith and clinical practice.

## Inclusion Criteria & Data Collection

To be considered for this study, participants had to have the title Music Therapist Accredited (MTA), be a member of the CAMT, be practicing members of an organized religion, and report themselves to be devout in their religious practice. Religious identities of established, organized religions were considered for this study. For example, atheism was not included as a religious practice given that “Atheism is not a belief system nor is it a religion” (American Atheists, n.d.). This criteria was in place for two reasons: 1) spiritualism seems to have gained wider acceptance in the therapeutic world due to its universal inclusion compared to the dogmatic beliefs of organized religions, and 2) one can belong to an organized religion without practicing the faith. Thus, it was necessary for the validity of this study to only include practicing members of organized religions in order to focus on the specific perspective. To be considered a practicing member of a religion, a participant had to actively participate in religious practices and customs of their faith (i.e., regularly attend religious services and observe essential dates) while also considering their religious beliefs to play a role in their day-to-day life.

**Figure 1.**
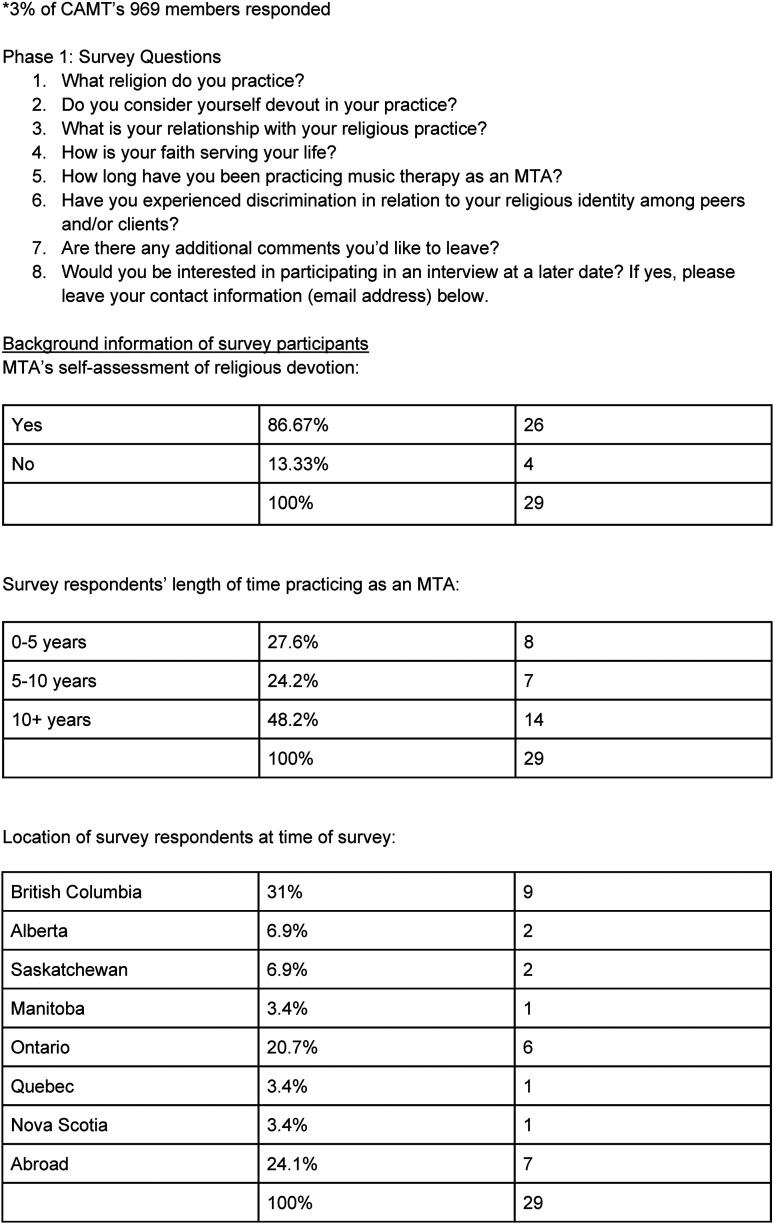
Survey distributed to members of the Canadian Association of Music Therapists (CAMT) from February 11–18, 2022, using Qualtrics.

The data collection took place over two phases. Phase one of the study collected data from an online survey generated by Qualtrics and was distributed by the CAMT to its 969 members (Canadian Association of Music Therapists, 2022a). The survey inquired about members’ religious faith, clinical practice, and experiences of perceived discrimination regarding their faith. The survey was completed by 29 members who fit the study's inclusion criteria and were located across Canada and abroad at the time of the survey. Respondents ranged from 0–10+ years as an accredited music therapist. Both the survey and in-depth qualitative data were collected through dichotomous and open questions (Figure 1). The survey was anonymous. However, respondents were allowed to leave a contact email address to be considered for phase two of the study.

**Figure 2.**
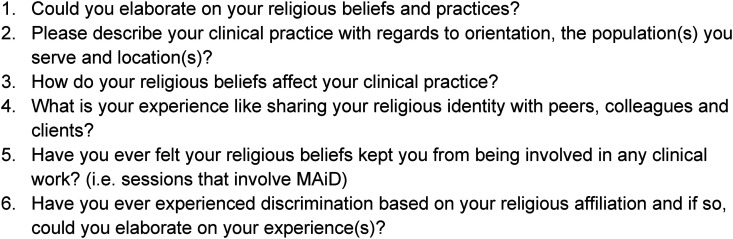
Phase 2: Interview Questions (Semi-Structured) - Conducted throughout March 2022.

The study's second phase was to collect data through in-depth, semi-structured interviews (Figure 2). Interview participants were selected based on their willingness to participate in an interview. Six survey respondents were contacted to participate in the second phase, and all six respondents accepted the invitation. Of the six participants, four identified as Christian, one as Baha'i and one as Jewish (Table 1). Regarding location, three participants were in British Columbia, two were in Ontario, and one was abroad. Individual interviews were conducted with the primary investigator during a 90-min video conference, with the option of having a follow-up conference if necessary. One participant took part in a 45-min follow-up interview. During the 90-min interview, participants were asked questions similar to the survey questions so that they could expand on their initial survey answers. A semi-structured interview approach was implemented so that participants had the freedom to express what they felt was specific and relevant to their lived experience. Video conferences were recorded and later transcribed.

**Table 1.** Religious affiliations of Phase 2 participants.

Participant 1 (P1)	Christian	Baptist
Participant 2 (P2)	Christian	Catholic
Participant 3 (P3)	Jewish	Reform
Participant 4 (P4)	Christian	Evangelical
Participant 5 (P5)	Baha'i	N/A
Participant 6 (P6)	Christian	Evangelical
		

**Figure 3.**
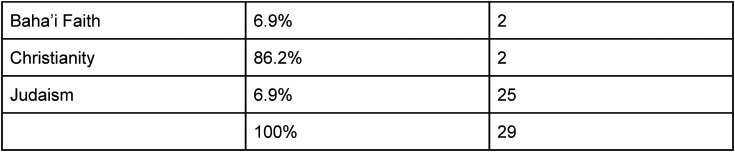
Religions practiced by survey respondents (based on 3% of CAMT's 969 members).

## Data Analysis

The qualitative data collected from the survey and interviews were analyzed using phenomenological thematic analysis through analytic labeling and dissected into categories of meaning. The interview transcripts were coded using NVivo software based on the themes presented and placed into categories for further analysis. The data was initially categorized into seven main categories: religious affiliation, populations worked with, modalities used, location and experience, faith's impact on clinical practice, faith and discrimination, and conflicts between faith and practice. Data was further dissected into subcategories within the categories to extract core meanings (Table 2). Abductive reasoning was used to extract and categorize data due to the influence of the researchers’ pre-existing knowledge on this topic (Peirce, 1903). Through this analysis, themes began to show themselves as having a positive impact (i.e., enhancements in clinical practice due to religious practice) and a negative impact (i.e., diminishments in clinical practice due to religious practice). Data was further processed using the Interpretive Phenomenological Analysis Model by Smith et al. (2009), during which “participants make sense of their experiences and the researcher, in turn, interprets the participants’ meaning-making to gain a fuller understanding of the experience” (Ghetti, 2016, p. 776). During this process, the data could speak, guide researchers’ understanding, and expand their perspectives through rereading and coding transcripts. The goal was to ensure that the possible insights were noticed and to regularly refer back to the research questions to maintain the study's direction. Lastly, the data was synthesized according to the research questions and the following three questions of Ansdell and Pavlicevic (2001, p. 1) Is this coherent and logical?; 2) Is this demonstrable?; and 3) Is this useful? Subsequently, findings were compared to previous research on this topic to determine if this study offered anything new.

**Table 2.** Phase 2 Coded Data.

Themes	Codes
Religious influence on clinical practice	Beliefs in an afterlife Sense of community Draw to music therapy as a career Clinical inspiration Clinical guidance Acts of service Prayer as a reflective practice Grounding/anchor Dignity at all phases of life Hope Peace and acceptance
Client population	Children (developmental disabilities, ASD, ADHD) Adult mental health Traumatic brain injury Dementia care Long term care Palliative care Bereavement
Modality	Person centered Humanistic Music centered Guided Imagery and Music (GIM) Community music-based model Neurologic music therapy
Religious devices used in clinical practice	Hymns with clients Prayer with clients Rituals with clients
Conflicts between faith and practice	When/how to share religious affiliation with clients/peers/colleagues How to incorporate religious practice in sessions when invited by client MAiD
Religious identity and perceived discrimination in workplace/academia	Job security Judgment from clients/peers/colleagues Feelings of unease from clients/peers/colleagues

## Results

**Figure 4.**
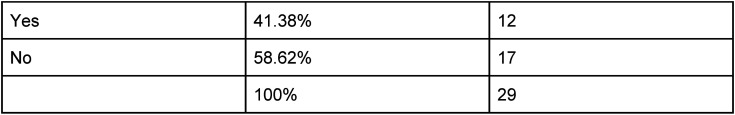
Experiences of perceived discrimination by survey respondents with colleagues, peers and/or clients in relation to religious affiliation.

**Figure 5.**
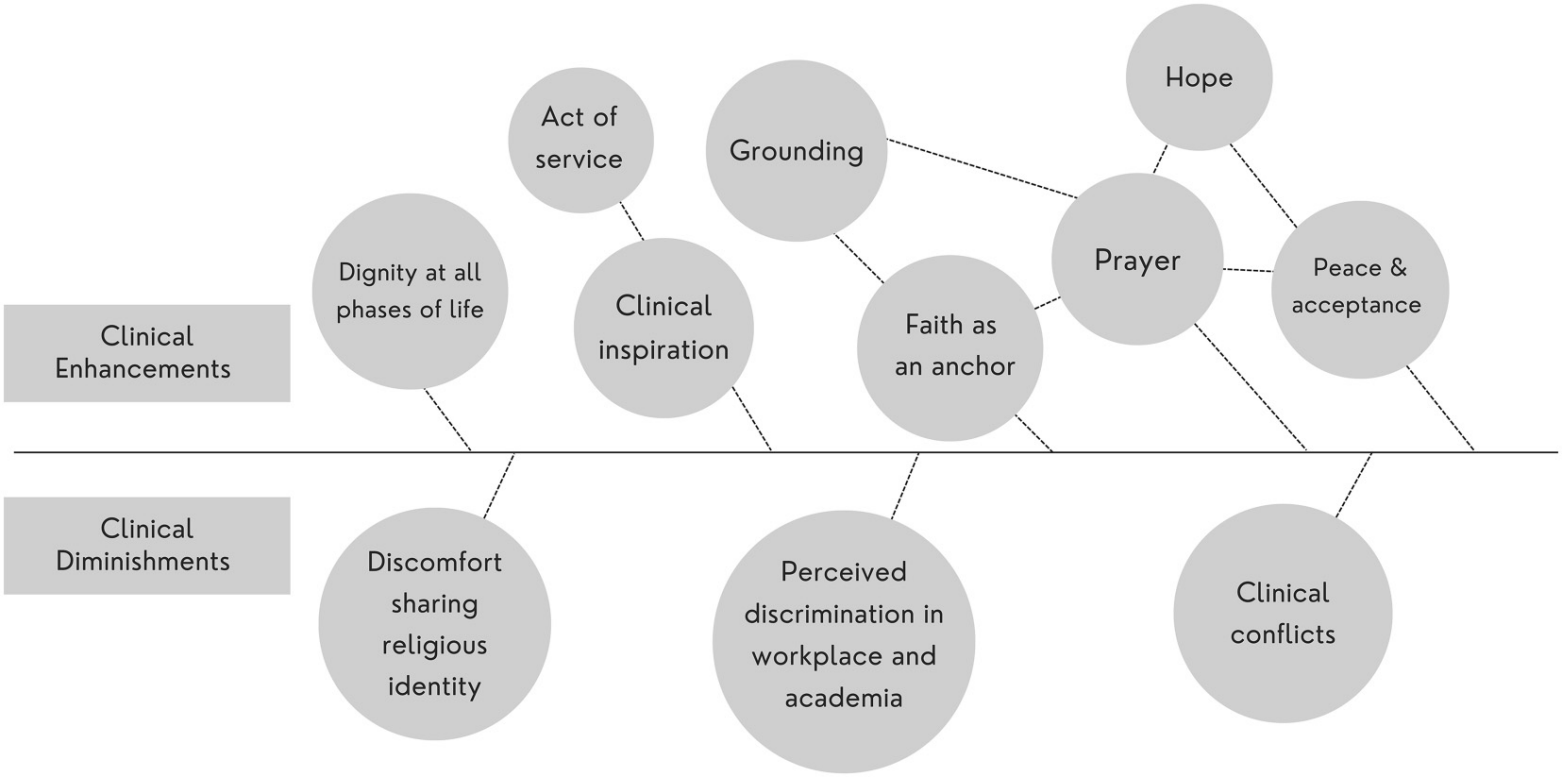
Data analysis from interview participants highlighting interconnectivity of themes and subthemes.

The survey distributed by the CAMT revealed 86.22% of the respondents identified as Christian, 6.89% identified as Jewish, and 6.89% identified as Baha’i (Figure 3). The survey also revealed 41.38% of respondents experienced some level of discrimination by colleagues, peers or clients about their religious affiliation (Figure 4). Data collected through the open survey questions and interview portion of the study suggested that belonging to and participating in religious practices contributed to their affirmation of pursuing music therapy, their faith acting as an anchor, gaining strength through prayer and maintaining emotional stability especially while clinicians navigated challenging client situations. Further, values present in their religious beliefs showed themselves in clinical work, such as offering compassion and dignity for all life. This was categorized as clinical enhancements directly linked to their religious practice. Half of the interview participants expressed experiencing some level of perceived discrimination about their religious affiliation. Most participants expressed discomfort sharing their religious beliefs with colleagues, peers, or clients. This was categorized as clinical diminishments due to their religious practice (Figure 5). In the following findings, quotations of interview participants have been lightly edited for conciseness and readability.

## Clinical Enhancements due to Religious Affiliation

### Clinical Inspiration and Music Therapy as an act of Service

Most interview participants reported a direct link between their religious affiliation, how they were drawn to music therapy, and how they are continuously inspired in their clinical practice. P2 reported, “The reason why I wanted to go into music therapy was because I was looking for a way to serve, which comes from wanting to love, wanting to share Christ's love, God's love with the world and looking for a unique way that I could do that using some of the gifts that He has given me.” A similar sentiment was shared by P5, who reported, “A big part of the Baha'i faith is service, so it's like finding what are your gifts God has given you, and how can you serve humanity using those gifts.” P5 also reported that they pursued the music therapy profession due to a series of occurrences as they discovered the Baha'i faith and that the two became closely linked.

Music therapists serve various populations across different levels of well-being and ill-health. Participants reported that their faith played a crucial role in their decisions to work with certain populations and their confidence in taking on challenging cases. P3 reflected on their conversion to Judaism and its connection to their clinical practice. P3 described Judaism as being an action-based religion and stated:

It is interesting, I’ve been thinking about this a little bit how what drew me to Judaism is sort of the same as what's drawing me to palliative care - the focus on the living and the focus on support for the living… I have also a very family centered approach and so when I'm working with a client who's dying I'm also working with the family. That in itself brings me comfort - you know the client has died, but I'm able to help the family still. I'm still very much in an action centered way [offering] things that I can do with the family to still bring comfort to them and then for myself.

Further, P4 reflected on working with a client who experienced a catastrophic brain injury and explained, “The report was so overwhelmingly challenging, and whether I should take it or not, and then, with my faith, I accepted the challenge… Long story short, it took a year and a half, but we had made some tremendous breakthroughs cognitively for that individual, and she was ultimately instrumental in steering me more towards brain trauma.” All participants seemed to find some direct inspiration from their religious practice that served as a continuous motivator and blueprint for how they carried out their clinical practice.

### Dignity for all Life

Many participants reported that their religious practice contributed to how they viewed and respected their clients. Participants described the notion of dignity as being a crucial aspect of the client/therapist relationship. In the Christian tradition, dignity at all stages of life is a core value embedded in teachings (Müller, 2020). When interview participants were asked how their religious practice influenced their clinical practice, the four Christian participants mentioned valuing life at all stages as a belief influencing their clinical work. P4 reported, “I would advocate for the very elderly who are cognitively impaired and completely dependent on care because I value life; I see it as a gift.” P1 similarly reported, “I value relationships with people of different life stages [and] ages.” P2 shared that this core belief has become a foundational aspect of their practice. P2 reported, “I have done sessions with clients who are completely unresponsive and have no idea if anything is getting through or not…What matters is that they’re still as dignified as I am; they’re still worthy of my time and attention just like anyone else.” P6 offered insight regarding her experience working with elderly clients. P6 reported:

The principles and values of my faith, I think, contribute to why those people matter - that they’re individuals created by God and He cares about them whether they do something good or whether they don’t do something good. It doesn’t matter - they’re valuable, they’re precious human beings and they matter even when they can’t speak anymore or even when they’re having really difficult behaviors.

### Faith as an Anchor/Grounding Tool

All interview participants reported that their religious practice provided grounding and was an anchor, especially during challenging clinical situations. P3 reported, “Having faith is like having an open door you can go through if needed. It's like having a friend there that's not pushing themselves on you; you can sort of lean on them if you need it.” Further, P4 shared, “[My faith] anchors me, it steers me… Sometimes I look back and am surprised at how grounded I can be in those [difficult] situations because sometimes it was intense…” In connection to the grounding effect participants described, participants spoke of their religious communities, which were cited by all as an important aspect of their grounding. Participants’ religious communities were described as a force that created accountability, providing a sense of anchoring and a feeling of being understood. All participants also reported prayer as a significant and effective tool for grounding and practice.

### Prayer

All participants described having a prayer practice that allowed them to reflect, connect and find refuge from their clinical work and was reported as central to their religious practice. P6 reported, “I think that practices of prayer and meditation have kind of led to an ability to go to that place even in the middle of a workday…. a space of prayer or acknowledgement of God or something like that, and I feel like that is something that can help me throughout my day…” Prayer was also described as relieving anxiety and maintaining assurance between the self and a higher power. P5 described, “I think [prayer] probably calms me down a bit… because when I'm praying at home, my nervous system knows that I'm calm and I'm connected to that force, that higher power - so I think it does help me stay grounded.” Prayer was cited as a tool to reflect and seek guidance in challenging clinical situations. P4 reported, “I pray for wisdom to know what to do, I pray to know what to say and what not to say because I'm dealing with crises [in] most of my clinical work… and that is grounding for me.” Lastly, all participants shared at least one instance where they privately prayed for a client, with some reporting that their clients have asked them to pray for them. P5 shared, “Praying for clients, especially those that have passed on - I did quite a bit of work in long-term care and always, even if it's just a short prayer, always pray for their soul, like the progress of their soul.” Participants reported that they would not initiate prayer with a client. However, participants reported that there have been instances where clients have asked them to pray on their behalf. When asked this by a client, P4 explained, “I will tell them that I will because the client asked me if I would just the same as they might have asked me to get a glass of water or to go out and call the nurse.”

### Peace and Acceptance

Religious practices assisted participants in finding peace and acceptance during difficult clinical situations. In populations where death was a regular part of the clinical experience, such as in palliative care or where catastrophic events resulted in life-changing circumstances, participants reported being able to accept complex outcomes. P2 reported, “Especially in palliative care and the way that Catholicism approaches death and views death has been very helpful for me because there's not a stigma around death… We gather, we honor, and we talk about our dead, and there's the belief in the afterlife and the hope of resurrection, so it becomes much less of a scary thing.” P2 also made reference to death being a transition to “going home,” which they explained was a common Catholic reference regarding one's passing. P2 explained, “It can be a really beautiful, peaceful thing you know, to be going home essentially, and so I think that is a huge part of what drew me towards that work at all, with that view - that part of life is beautiful.” P6 also shared how they cope during end-of-life care through their faith. P6 shared, “When a client passes away, I think that their suffering is over; I think that they can be experiencing togetherness with and love with God, in whatever that looks like… It helps me to have some comfort and some peace, even if I do feel sad or some anger about the circumstances of a person's passing.” Participants reported that although they have experienced sadness in their clinical work, it has never been debilitating or unmanageable. P4 reported, “I have grieved, but I've not been distraught,” and further explained, “I do have a strong sense of peace, I feel anchored… I'm not afraid; I'm not worried about death; I have a real sense of peace.”

### Hope

Lastly, many participants reported that their religious beliefs were a source of hope in their clinical work and assisted in their emotional stamina to engage in complex clinical situations. P4 reported, “I go in with hope, and that has given me the strength to hang in tough cases or deal with emotionally tense situations with that patient or their family.” P4 continued, “I do not go in thinking that I'm going to make amazing progress because they may not make amazing progress, but I have hope for some shift in a positive direction so if I see any progress, that's success…” Hope was also described as being closely linked to feeling grounded, as was reported by P1. P1 shared, “Part of the grounding I think is hope, like where we find hope, and we talked about that a lot in our community - that's [been] an important piece, especially during the pandemic.” Hope seemed to be a byproduct of religious practice and instrumental in serving as clinical motivation.

## Clinical Diminishments due to Religious Affiliation

### Discomfort Sharing Religious Identity

All participants reported discomfort when sharing their religious affiliation with colleagues, peers or clients. Some chose not to disclose for fear of negative repercussions directly. Although some Christian participants expressed the historical privilege of practicing Christianity in Canada, all expressed fear of being pigeonholed into Christian stereotypes. P6 shared, “I am unfairly painted with the same brush as the worst and most destructive members of Christianity, past and present, and I do not enjoy how it feels…” All Christian participants also acknowledged problematic historical events of their faith and its resulting trauma, thus playing a role in their decision to disclose. P1 shared, “I want to be as open and inclusive as possible, and I'm sensitive that people can be triggered by or have baggage around Christian faiths because it's so prominent…” P3 shared that a deterrent was fear of experiencing anti-semitism in the workplace, something that they had experienced outside of the workplace. P3 reported, “I think I'm pretty careful not to say that I'm Jewish… I don't want it to impact the [therapeutic] relationship - which is really sad because I feel like a lot of other people of different faiths don't have to think about that.” Although P5 expressed no trepidation in disclosing their faith identity, misconceptions of their religious affiliation were cited as a source of discomfort. P5 reflected on a staff meeting where staff was encouraged to share a personal item, “I was sharing my Baha'i prayer book because none of my colleagues knew that I was Baha'i, and it was kind of scary because working in an organization that serves sex workers, there is a Christian organization in town that does not support sex workers and is actually harmful [to them] so I had this fear that they would put me in this category with this Christian group.” P5 reported that their religious practice was received warmly and became an opportunity to educate colleagues about their faith. All participants expressed that discretion was used when and if they considered sharing their religious identity with clients, with the primary factor being if it was beneficial to the client to self-disclose.

### Perceived Discrimination in Workplace and Academia

Perceived discrimination describes the lived experience by those encountering bias in everyday attitudes subtly relating to their specific characteristics (Banks et al., 2006). Half of the interview participants reported instances of perceived discrimination in either the workplace or in academia. P6 reported:

Though I typically don't disclose my faith background to clients, those who witness me leading spiritual programs seem to open up to me less, and occasionally make subtle comments alluding to their distaste or distrust of the religion - understandable, in my opinion. Colleagues have occasionally made jokes regarding my faith, have been less relationally open with me, unnecessarily apologize when speaking freely, and even sometimes speak openly negatively in my presence about the faith which I practice.

P2 reported:

There have been no overt instances of discrimination, however I have often experienced a generalized negativity toward my beliefs and my association with the Catholic Church. I have had clients speak negatively about the church to me, essentially calling Catholics “stupid.” And I have had many peers express animosity toward Catholics to the point where I become extremely anxious and feel as though I cannot disclose my beliefs or opinions.

With regards to academia, P4 reported:

The dominant attitude that's projected in the classes… is that if you are highly educated, you would not embrace Christianity because postmodernism, open mindedness and advanced thinking would reject that. And in fact, Christianity is blamed for a lot of the world's and society's [problems] past and present. So the logical conclusion to that is, if you do express that you are Christian, you are perceived as being less intelligent, narrow minded, probably biased against half of the earth and shouldn’t have a voice in case you’re trying to recruit people for your faith.

Participants who reported instances of perceived discrimination also described subsequent emotional distress. Participants described stress, anxiety, sadness and overall discomfort about the diminishments their religious affiliation placed on their clinical life. Coping strategies to alleviate the distress reported to be prayer and taking opportunities to use their voice appropriately and respectfully.

### Clinical Conflicts

Clinical conflicts between religious affiliation and clinical practice show themselves when an internal dissonance is centered around one's values and how they affect clinical practice. A common conflict that participants faced was determining how to navigate spirituality as a clinical practice component. Especially for those working in palliative care, the desire to help clients cope in these settings while not incorporating their own beliefs was described as an initial conflict. P4 reflected on their work in palliative care and explained “the urgency, sometimes of pending death and that person needing spiritual counsel.” P4 reported:

I was not there in that role, and that was very, very hard. My solution was I found a couple of local pastors who were willing to be called last minute. And so, when spiritual concerns came up for a client in palliative care and my role as a music therapist didn't permit me to explore them… [I made] a referral system to fill that role.

P4 continues, “That was an ethical dilemma for me because I would like to have addressed the spiritual, but you are not supposed to express your own opinions on these things as a music therapist because it may look like you're recruiting or trying to sway someone.” Other examples showed itself in music therapy's role in medical procedures. P2 explained their moral struggle when working in palliative care and clients undergoing Medical Assistance in Dying (MAiD). MAiD was legalized in Canada in 2015, and music therapists, especially those working in palliative care, have become involved at their client's request (Black et al., 2020). P2 reported, “It was a moral struggle for sure…I believe that if we cannot honour our client's request, we should be referring to another music therapist, which in and of itself, at least in my faith, you would still be considered complicit.” This moral struggle ultimately resulted in this participant leaving palliative care to alleviate the internal dissonance experienced between their religious faith and clinical practice. P2 also reported job security concerns due to their religious beliefs if they ever wanted to return to palliative and end-of-life care in the future. Other participants explained their application of compartmentalization about their religious beliefs and clinical work. P3 shared, “You sort of have to divide yourself a little bit because you want to be there in the best interest of your clients.” All participants expressed the importance of acting within the client's best interest, whether separating select religious beliefs from their clinical practice or removing themselves from the therapeutic relationship.

## Discussion

This study revealed two main categories that present themselves in clinical practice due to being a religiously devout music therapist: clinical enhancements and clinical diminishments. While only three religions were represented in this study, the data from each interview participant displayed more similarities than differences. All interview participants reported that their faith was an anchor that grounded them in their clinical work. All used prayer as a form of reflective practice and utilized elements of their faith as a blueprint for navigating their clinical life. Suppose this study was expanded to include music therapists of other religious affiliations. In that case, it begs whether there would be significant changes to the data given the many similarities world religions share. The Dalai Lama has stated, “All the major religions of the world have similar ideals of love, the same goal of benefiting humanity through spiritual practice, and the same effect of making their followers into better human beings” (Templeton, 1999, p. 2). The accuracy of this statement can be seen in the religions examined in this study. Such can be categorized as the clinical enhancements a clinician can experience through religious practice. Of the two main categories examined in this study, clinical diminishments may be the area that reveals the most significant differences between religious affiliations, given the societal interpretations of varying religions and other factors. For example, experiences of perceived discrimination may have higher instances in certain religious practices than others, and how this affects a clinician's practice could vary.

When examining the diversity among the music therapist population in Canada, it is essential to reference the country's religious makeup. In recent decades, Canada has been viewed as a cultural mosaic with a wide range of diversity in ethnicities and faiths. Although this is true in urban areas, the rural areas which make up most of Canada's landmass are predominantly Christian. In referencing the religious makeup of Canada, a survey by Statistics Canada between 2017 and 2019 revealed that 63.2% of the population were Christian (all denominations), 1% Jewish and 1% Other (including the Baha'i faith), which is comparable to the statistics found in this study's survey (Cornelissen, 2021). The lack of diversity in this study could be due to the religious landscape within Canada and the already small number of MTAs.

Of the religious faiths examined in this study, only Christian music therapists reported instances of perceived discrimination. With these admissions, many of the music therapists emphasized their acknowledgment of dark historical events of Christianity, and some felt their feelings of discrimination were not warranted because of this. Christianity's role in Canadian residential schools and the recent discovery of Indigenous children's unmarked graves (Cooper, 2022) are just an example of the moral vicissitudes of some Christian denominations. In addition to the seemingly problematic pasts of many world religions, religions tend to be exclusive and can be discriminatory in certain aspects. For example, in the Baha'i faith, marriage is only permitted between a man and a woman (Hartz, 2009), and this is also true for most Christian and Jewish faiths. The treatment of LGBTQ+ communities continues to be discriminatory at the hands of many world religions, and it is because of these non-inclusive beliefs in the religious practices that the participants may have found difficulty in claiming their own discriminative experiences.

Although some participants reported experiences of perceived discrimination, all participants expressed discomfort in sharing their religious affiliation with peers, clients or peers in fear of experiencing negative repercussions. Participants listed being associated with negative stereotypes and being seen as less intelligent as examples of negative repercussions. This raises important implications for the ways in which the music therapy community embraces members of varying religious beliefs in the same way they are encouraged to embrace clients’ beliefs. Referencing the Canadian Charter of Rights and Freedoms (1982) under Section 2a, freedom of religion is described as “the right to entertain such religious beliefs as a person chooses, the right to declare religious beliefs openly and without fear of hindrance or reprisal, and the right to manifest religious belief by worship and practice or by teaching and dissemination” (Government of Canada, 2022). In theory, Canadians can practice their religion without fear of reprisal. However, religion remains a guarded aspect of one's identity, and as such, one may be reticent about exposing one's religious affiliation in a clinical setting.

Another aspect that showed itself in the data was the inner conflict of incorporating spiritual elements into the music therapy session. If we look to our professional association for guidance on the integration of spiritual practice in sessions, the CAMT lists “spiritual domains” as an area that can be addressed within music therapy in their Code of Ethics (p.4) and although the document does not offer specific guidelines on how to integrate this component safely, it does state a music therapist must, “only provide services and use music therapy approaches for which they have established competence through pre-professional or advanced training and supervision” in Code 2.4 (Canadian Association of Music Therapists, 2022b, p.8). Incorporating spirituality in music therapy raises important implications and ethical considerations for the music therapist. A study by Masko (2016) explored the perspectives on ethics and training issues of music therapists working in hospice care. The study found that music therapists feel responsible for implementing spirituality in their practice with hospice patients. However, they feel they need adequate training and support to do so.

Further, The CAMT Code of Ethics makes mention of “religious beliefs” (among other areas) as prohibited grounds for discrimination including remarks, jokes etc. (Canadian Association of Music Therapists, 2022b, p 5). As the data from this study reveals, some interview participants reported instances of remarks, jokes and general unease in relation to their faith practice in a work/academic setting. Although the Code acknowledges religious beliefs as an area requiring respect, it does not speak directly to conflicts in belief systems a music therapist might experience (for example, involvement in MAiD). However, they state that, “The MTA will refer clients to other Certified Music Therapists or professionals when the client requires or requests services that are beyond the music therapist's established level of competency or outside the music therapy scope of practice” in Code 2.7 (Canadian Association of Music Therapists, 2022b, p 8).

By interviewing six music therapists representing different religions, parts of Canada, and populations they worked with, the findings reflect multiple perspectives and realities, albeit from a minimal sample size. Data was interpreted using disciplined subjectivity so that it was possible to reflect critically on researchers’ own interpretations—for example, as a practicing Catholic and a student music therapist at the time of this study, Victoria aimed to avoid biases that could have presented themselves, and through disciplined subjectivity, she hoped to mitigate this.

## Ethics & Limitations

This study was approved by the Research Ethics Board at Wilfrid Laurier University, REB # 7083, and all participants provided informed consent. The study has the following limitations, and the authors highlight further avenues of exploration.

Due to the minor nature of this study, the data reflects only 3% of the CAMT members and represents three organized religions. Given this small sample size and the underrepresentation of other organized religions, the findings could be limited to the participants and not be a broad representation of the relationship between religious faith and clinical practice. Similarly, the limited participants in the study cannot represent each faith as a whole since every individual has their own experiences and interpretations that influence how they practice their faith. Further, the faith practices examined in this study are theistic, and the researchers were unable to include music therapists from non-theistic religious traditions. Thus, the findings, such as using prayer to a higher power for reassurance and guidance in clinical practice, may not apply to those of non-theistic faiths. As these are limitations of the study, we hope future studies with more participants and more diverse faiths can continue to investigate the relationship between religious faith and clinical practice.

## Conclusion

This study investigated how religious beliefs and practices affect a music therapist's identity and clinical practice. Further, this study sheds light on the benefits religious practice lends to clinical work while also revealing disparities present in the field of music therapy among religiously devout clinicians. A survey distributed by the CAMT garnered responses from twenty-nine members, six of whom were contacted to participate in semi-structured interviews. The survey participants examined their religious practices within three organized religions: Baha’i, Christianity, and Judaism, with interview participants representing these faith traditions. Findings from this study revealed two main categories impacting clinical practice: clinical enhancements and clinical diminishments. Examples of clinical enhancements include framing clinical work as an act of service, using prayer as a tool to navigate challenging clinical situations, and faith acting as a grounding force in their daily clinical practice. Contrarily, examples of clinical diminishments include discomfort in disclosing religious affiliations to colleagues, peers, or clients and perceived discrimination in the workplace. Although all interview participants described their religious affiliations as having positive effects on their practice, all participants described some level of discomfort in openly expressing their religious identities in fear of experiencing negative repercussions by colleagues, peers or clients.

While three organized religions were investigated in this study, the data from interview participants appeared to have more similarities than differences. All interview participants reported that their faith served as an anchor that grounded them in their clinical work. Further, all interview participants cited using prayer as a form of reflective practice while applying elements of their faith as a blueprint for navigating their clinical life. Although clinical enhancements gathered the most similarities, the clinical diminishments found the most differences. This can be seen when exploring experiences of perceived discrimination, as there were higher instances of discrimination in certain religious practices than in others. Thus, the social perception of specific faith practices could affect how clinicians embrace their religious faith alongside clinical practice.

Another aspect highlighted among interview participants was the uncertainty of incorporating faith-based practices within sessions. It is understood that a therapist's self-disclosure and safe and effective use of self can strengthen the therapeutic alliance. However, the approach to incorporating religious faith into clinical practice seems unclear. Traditionally, it is encouraged that the psychotherapy session remains secular. However, this can directly contradict spiritual well-being, a goal commonly worked towards in music therapy practice. The spiritual domain is listed as a potential area of exploration in music therapy by the CAMT, though navigating this within a standardized approach seemed challenging among participants.

Given the small sample size and underrepresentation of organized religions, further international research should be conducted on the topic to better understand the relationship between clinical practice and religious devotion. As this study was conducted in Canada, a country with a predominantly Christian population, a globalized study may draw more diverse results and further insight into how religious devotion affects a music therapist's clinical practice. Although the clinical enhancements revealed in this study seemed to have similarities across the different organized religions, disparities seemed to be highlighted among the clinical diminishments. Further research on this topic is encouraged as investigating more diverse religious practices may paint a clearer picture of the impact religious practice has on music therapy practice. 
